# Application of Network Analysis to Flow Systems with Alternating Wave Channels: Part B. (Superimposed Drag-Pressure Flows in Extrusion)

**DOI:** 10.3390/polym12091900

**Published:** 2020-08-24

**Authors:** Christian Marschik, Wolfgang Roland, Marius Dörner, Sarah Schaufler, Volker Schöppner, Georg Steinbichler

**Affiliations:** 1 Institute of Polymer Extrusion and Compounding, Johannes Kepler University Linz, 4040 Linz, Austria; wolfgang.roland@jku.at (W.R.); sarah.schaufler@jku.at (S.S.); georg.steinbichler@jku.at (G.S.); 2 Kunststofftechnik Paderborn, Paderborn University, 33098 Paderborn, Germany; marius.doerner@ktp.uni-paderborn.de (M.D.); volker.schoeppner@ktp.uni-paderborn.de (V.S.)

**Keywords:** wave-dispersion screw, modeling and simulation, polymer processing, extrusion, network theory

## Abstract

Due to progress in the development of screw designs over recent decades, numerous high-performance screws have become commercially available in single-screw extrusion. While some of these advanced designs have been studied intensively, others have received comparatively less attention. We developed and validated a semi-numerical network-theory-based modeling approach to predicting flows of shear-thinning polymer melts in wave-dispersion screws. In the first part (Part A), we systematically reduced the complexity of the flow analysis by omitting the influence of the screw rotation on the conveying behavior of the wave zone. In this part (Part B), we extended the original theory by considering the drag flow imposed by the screw. Two- and three-dimensional melt-conveying models were combined to predict locally the conveying characteristics of the wave channels in a discretized flow network. Extensive experiments were performed on a laboratory single-screw extruder, using various barrel designs and wave-dispersion screws. The predictions of our semi-numerical modeling approach for the axial pressure profile along the wave-dispersion zone accurately reproduce the experimental data. Removing the need for time-consuming numerical simulations, this modeling approach enables fast analyses of the conveying behavior of wave-dispersion zones, thereby offering a useful tool for design and optimization studies and process troubleshooting.

## 1. Introduction

From a quantitative point of view, extrusion is the most important process in the polymer industry. Every year, this technique converts more than 114 million tons of polymeric materials to products ranging from sheets to profiles and from pipes to films [1]. To meet the ever-increasing demands of the plastics industry, a trend towards use of high-output extruders has emerged over recent decades. These developments have also resulted in a variety of high-performance screws being designed to provide excellent melt homogeneity at high output rates and proper discharge temperatures. Despite their importance, only a few studies have examined the performance of wave-dispersion screws [2,3,4,5,6].

The concept of wave-dispersion screws was initially proposed by Kruder [7]: Replacing the metering zone of conventional extruder screws, a so-called wave section is implemented in which the channel depth oscillates over a plurality of cycles. Assuming the resin to be mostly in a molten state, the deep valley portions minimize heat generation in the screw channel, while the shallow peak portions ensure repeated intensive mixing.

The efficiency of the originally proposed concept depends strongly on the solids content in the extrudate, as solid resin particles may restrict the flow path at the wave peaks, causing the extrusion process to become unstable. To improve the performance, Kruder [8] developed the double-wave screw. This term refers to wave sections consisting of two parallel screw channels separated by a barrier flight that is undercut relative to the main flight. The wave cycles in the adjacent channels are out of phase, which means that, as one sub-channel decreases in depth, the other sub-channel increases. The helical offset is typically arranged such that a wave peak of one channel lies opposite a valley in the other and vice versa. In contrast to the original single-flighted design, this concept allows the polymer melt approaching a wave peak to pass across the undercut barrier, thereby minimizing the risk of blockage and pressure fluctuations. With the material being repeatedly transferred between the channels, the screw design promotes cross-channel mixing and improves melt homogeneity.

A modified concept of the double-wave screw, trademarked as energy-transfer screw, was developed by Chung and Barr [9]. In their design, the clearances of the screw flights are selectively interrupted such that the functions of the main and the secondary flights alternate. In contrast to the double-wave screw, in which the material is conveyed within the same pair of subchannels, the energy-transfer screw allows the material to flow only in the direction of the natural flow caused by the rotation of the screw. While this concept once again improves the mixing performance of the screw, it can limit the pressure build-up capacity. Several wave-dispersion screw patents followed these developments [10,11,12,13].

Due to their great mixing capability, wave-dispersion zones were originally located at the discharge end of the extruder. In recent years, the geometrical design was extended to the melting zone to break up solid agglomerates and mix it with molten material in the screw channel.

### 1.1. Analysis of Melt Conveying and Pressurization

The analysis of polymer-melt flow in single-screw extruders has been the subject of numerous theoretical studies. Most of these examined the flow in conventional metering sections, whereas wave-dispersion zones received less attention in comparison. The first model of melt conveying in single-screw extruders was published anonymously [14] and later extended by several studies [15,16,17,18]. These early analyses investigated the flow of a temperature-independent Newtonian fluid and provided exact analytical solutions for the drag- and pressure flows in the cross- and down-channel directions.

Taking the shear-thinning flow behavior of polymer melt into account increases the complexity substantially. Due to the dependency of viscosity on shear rate, the flow components are coupled, and even for a one-dimensional flow of a power-law fluid, exact closed-form analytical solutions remain elusive. To extend the flow analyses to shear-thinning fluids, numerical solutions have been presented for one- and two-dimensional flows [19,20,21,22]. The analysis gains an additional degree of complexity when transverse flow is included, which contributes to the magnitude of shear rate and to the viscosity of the polymer melt, thus indirectly affecting the flow rate. This effect is particularly pronounced in the case of wave-dispersion screws, where cross-channel mixing is systematically increased by the geometry of the wave channels.

With the advent of more advanced computers, numerous works [23,24,25,26,27] addressed full three-dimensional flows in conventional metering channels using methods based on computational fluid dynamics (CFD). By numerically solving the governing flow equations, these analyses provided detailed insights into the velocity and pressure characteristics of the recirculatory flow. Numerical simulations have also proven useful in predicting the conveying behavior of wave-dispersion screws [2,3,4]. Due to the increased complexity of the flow situation and the limited computational power at the time, only a few channel geometries and physical conditions were considered.

To estimate the effect of shear-thinning flow behavior on the pumping capability of conventional metering zones, several studies proposed analytical regression models [28,29,30,31,32,33]. Approximating the numerical results of numerous flow simulations, these melt-conveying models were designed to predict the throughput as a function of characteristic process parameters without resorting to numerical methods.

### 1.2. Research Approach

**Figure 1:**
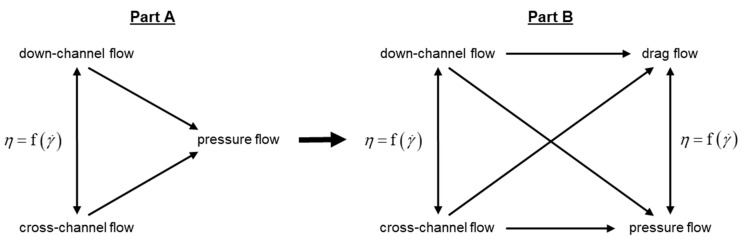
Research approach: In the first part (Part A), the pressure flow was decoupled from the drag flow. With the shear-thinning flow behavior being considered, a coupled network of down- and cross-channel flows was solved. In this part (Part B), the influence of the rotation is added.

Due to the lack of analytical melt-conveying models available for wave-dispersion screws and to remove the need for computationally expensive and time-consuming CFD simulations, we propose a semi-numerical modeling approach to predicting the flow of shear-thinning polymer melts in wave-dispersion zones. Rather than numerically solving a full set of conservation and constitutive equations, our semi-numerical method iteratively solves a linearized set of network equations and is therefore considerably faster. Figure 1 illustrates the main characteristics of the research approach, which was split into two parts (Part A and Part B).

In Part A [34], we systematically reduced the complexity of the flow analysis by ignoring the influence of screw rotation on the conveying behavior of the wave zone. Focusing on three-dimensional pressure flows allowed us to develop and validate a semi-numerical modeling approach to predicting (i) the pressure distribution, (ii) the local down- and cross-channel flows, and (iii) the transverse mixing capability in double-wave-channel systems.

In this part, we incorporated the influence of screw rotation into the modeling procedure. To this end, two- and three-dimensional melt-conveying models, presented in [30,31,32], were used in the network calculation, replacing the governing pressure-flow equation in the original theory. Moreover, the structure of the flow network was modified to additionally include leakage flow over the main flights. Experimental studies were carried out using 35 mm single-screw extruders equipped with various barrels and double-wave screws. The results of the experiments were compared to those of our extended modeling approach.

## 2. Experimental Procedure

### 2.1. Materials

As in the first part of this research, two materials were analyzed: (i) a high-density polyethylene (melt-flow rate of 0.24 g/10 min measured at 190 °C/5 kg) and (ii) a polypropylene random copolymer (melt-flow rate of 8.0 g/10 min measured at 230 °C/2.16 kg). The first material (HDPE) is used in the manufacturing of pipes, whereas the second (PP-R) is applied in industrial film extrusion. The rheological flow behavior of the polymer melts was approximated by a Carreau-Yasuda model:

(1) $$\eta_{c}=a_{t}\eta_{\infty }+a_{t}\left(\eta_{0}-\eta_{\infty }\right){\left(1+{\left(a_{t}\;\lambda \;\dot{\gamma }\right)}^{a}\right)}^{\frac{n_{c}-1}{a}}$$

where $\eta_{0}$ and $\eta_{\infty }$ are the zero- and infinite-shear viscosity, respectively, λ is the characteristic relaxation time, and $n_{c}$ is the Carreau-Yasuda power-law index. The temperature-shift factor was evaluated by an approximated Arrhenius model:

(2) $$a_{t}=exp\left(-\alpha \left(T-T_{0}\right)\right)$$

where α is the temperature coefficient and $T_{0}$ the reference temperature. Table 1 lists the Carreau-Yasuda parameters and the melt densities of the materials. For detailed information on data measurement, the reader is referred to Part A [34].

### 2.2. Equipment

**Figure 2:**
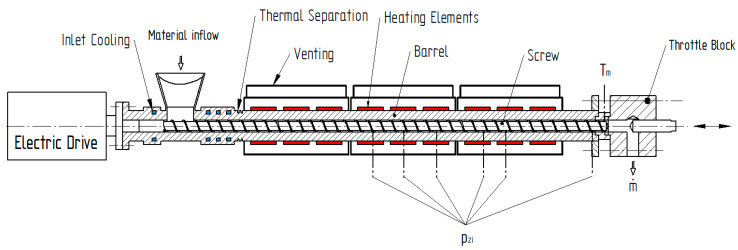
Schematic diagram of the 35 mm plasticating single-screw extruder.

Experimental studies were performed on a 35 mm SML single-screw extruder (SML, Lenzing, Austria). A schematic of the test setup is shown in Figure 2. The electrical drive unit provided a maximum mechanical power of 38.4 kW and a maximum screw speed of 434 rpm. At the extruder head, a throttle block equipped with an adjustable screw was installed to set the discharge pressure.

Along the extruder barrel, thermal energy was supplied by electrical heaters grouped into three heating zones: *T*_1_ to *T*_3_. Further, the temperature of the downstream threaded ring and the throttle block was controlled by two heating zones: *T*_4_ to *T*_5_. A water-cooled feed housing was used to avoid heat generation in the feeding zone, while the remaining barrel sections were cooled by forced air. Table 2 shows the temperature profiles used in the experimental part.

To allow various specific rates, we used three extruder barrels of diameter *D_b_* = 35 mm with different designs in the feeding zone of the screw. One of these was produced with an entirely smooth inner surface (barrel 1), whereas the others were constructed with short helical and axial grooves in the feeding zone (barrels 2 and 3). Table 3 lists characteristic geometrical properties of the barrels.

**Figure 3:**
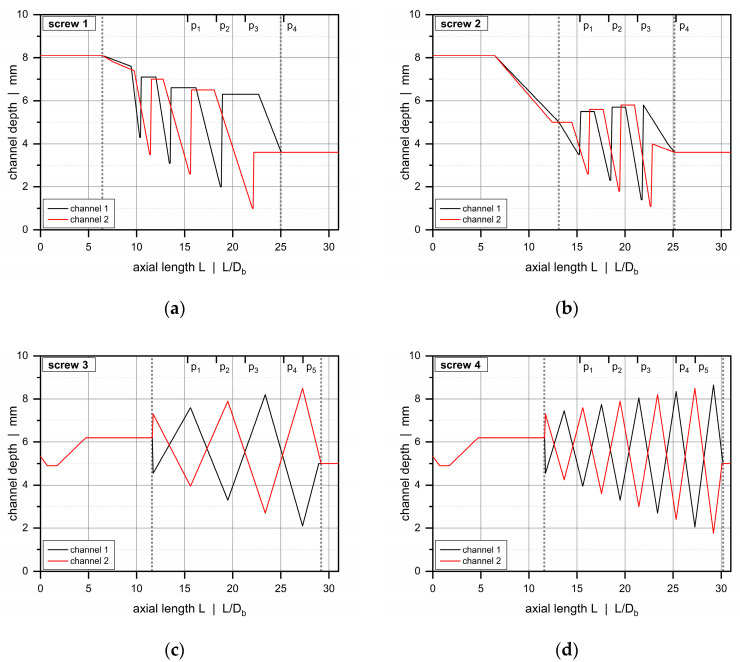
Comparison of channel-depth profiles: screw 1 (**a**), screw 2 (**b**), screw 3 (**c**), and screw 4 (**d**). The dotted vertical lines indicate the beginning and the end of the wave zone. The axial positions of the pressure transducers are shown at the upper x-axis.

For each barrel, we investigated two double-wave screws of outer diameter *D_S_* = 34.85 mm and axial length L = 1090 mm. In total, these involved two screws (screws 1 and 2) for the smooth-bore and two screws (screws 3 and 4) for the grooved-barrel configurations. To adapt the screw designs to the diverse conveying characteristics of conventional and feed-controlled extrusion processes, different screw geometries were used in each case. For each barrel system, however, the screws were identical except for the geometry of the wave zone. Figure 3 illustrates the channel-depth profiles of the screws.

For barrel 1, we used two double-wave screws with a feeding and a metering zone of channel depth *h_f_* = 8.1 mm and *h_m_* = 3.6 mm, respectively. The beginning of the wave zone was shifted from an axial position of 6.4 *D_b_* for screw 1 to 14.2 *D_b_* for screw 2, while the end of each wave zone was located roughly at the same axial position. The oscillating channel-depth profiles were constructed such that the numbers of wave peaks and crests were equal. The distance between the channel depth maxima, however, was decreased in the second case.

For barrels 2 and 3, the feeding zone of the double-wave screws was constructed with a compression from *h_f,_*_1_ = 4.9 mm to *h_f,_*_2_ = 6.2 mm, and the wave zone was extended to the end of the screw. In contrast to screws 1 and 2, the wave profiles showed a fully alternating behavior over the entire length of the zone. By changing the frequencies of the channel depth maxima, the number of wave peaks and valleys was almost doubled for screw 4.

For all double-wave screws, a Maddock shearing element with axial length *L* = 165 mm was employed. Table 4 compares geometrical parameters of the double-wave zones analyzed.

We positioned six piezoresistive pressure transducers along the barrel to measure the pressure profile along the screw. Due to the individual designs of the screws, the wave zones included four pressure sensors ($p_{1}$ to $p_{4}$) in the cases of screws 1 and 2, and five pressure sensors ($p_{1}$ to $p_{5}$) in the cases of screws 3 and 4 (Figure 3). The last pressure transducer $p_{6}$ was positioned at the screw tip to measure the discharge pressure. Axial positions, measuring ranges, and response times of the pressure sensors are summarized in Table 5. Further, the temperature of the polymer melt at the discharge end of the extruder was evaluated by a melt-temperature sensor.

### 2.3. Procedure

For each screw-barrel configuration and material, experiments were performed by increasing the screw speed from 25 rpm up to 400 rpm. Due to mechanical limitations of the barrels, we were not able to reach the maximum screw speed in all experimental setups. With the single-screw extruder operating at steady state, the throughput, the axial pressure profile, and the melt temperature were measured using a data acquisition unit. For each operating point, the processing parameters were recorded for one minute. Mean and standard deviation were evaluated to determine parameter fluctuations and the stability of the process. Table A1 in the Appendix A summarizes the experimental data for each operating point.

At the beginning of each test run, the discharge pressure was set to 250 bar for the HDPE and to 150 bar for the PP-R by changing the position of the screw mounted within the throttle block. These values represent characteristic settings found in pipe and film extrusion. The discharge pressure was adjusted once before each test at predefined screw speeds. During the experimental procedure, the position of the throttle screw was kept constant.

**Figure 4:**
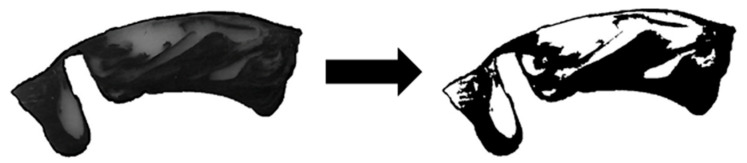
Solidification experiment: The solids and melt content along the wave zone, indicated by the white and black sections, respectively, were determined by image analysis.

In addition, we carried out solidification experiments for selected processing conditions using the HDPE to analyze the melting behavior of the screws. To this end, carbon black masterbatch was added to the white polymeric material, the extrusion process was run in stationary mode and then stopped abruptly, cooling down the extruder and thus solidifying the polymer melt. After pulling the screw from the processing unit, thin sections of the solidified material were removed from the helical ribbon. The solids and melt content along the wave zones were evaluated by image analysis. The white and black sections indicated solid material and polymer melt mixed with the masterbatch, respectively (Figure 4). Taking several samples from various axial positions, we assessed the melting progress by relating the area of the black spots to the total area of the cross section.

## 3. Modeling

### 3.1. Melt-Conveying Models

A novel feature of the theory presented here is the integration of two- and three-dimensional melt-conveying models (proposed in [30,31,32]) into the network calculation to describe the local conveying characteristics of the wave zone.

**Figure 5:**
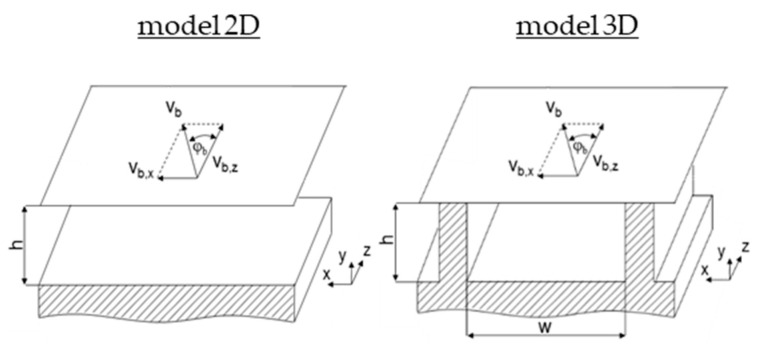
Representation of the screw channels in the two- and three-dimensional melt-conveying models. Ignoring the curvature of the screw, the flat-plate model with moving barrel was applied in both cases.

Replacing the governing pressure-flow equation in the original theory, these models predict fully developed isothermal flows of wall-adhering power-law fluids in two- and three-dimensional screw channels, as shown in Figure 5. The former screw channel is infinitely wide and the corresponding flow field shows velocity components in the cross- and down-channel directions. The latter screw channel includes the influence of the screw flights, thereby producing velocity components in all directions of the coordinate system.

The melt-conveying models were developed by means of a hybrid modeling procedure that incorporates analytical, numerical, and data-based modeling into one approach. Detailed information is given in [33]. For each flow situation, the governing flow equations were rewritten in dimensionless form to determine the independent dimensionless input parameters. These quantities were then varied to create a large set of physically independent design points, whose target variables were evaluated numerically. The numerical results of the parametric design studies were then approximated using symbolic regression based on genetic programming. The hybrid modeling approach yielded two analytical regression models that predict a dimensionless volume flow rate $\Pi_{V}$ as a function of the corresponding sets of independent dimensionless input parameters:

(3) $$2D:\;\;\;\;\;\Pi_{V}=f\left(\frac{t}{D_{b}},\;\;\;\;\;\;\;\;n,\;\Pi_{p,z}\right)$$

(4) $$3D:\;\;\;\;\;\Pi_{V}=f\left(\frac{h}{w},\;\;\;\;\;\;\;\;\frac{t}{D_{b}},\;n,\;\Pi_{p,z}\right)$$

**Figure 6:**
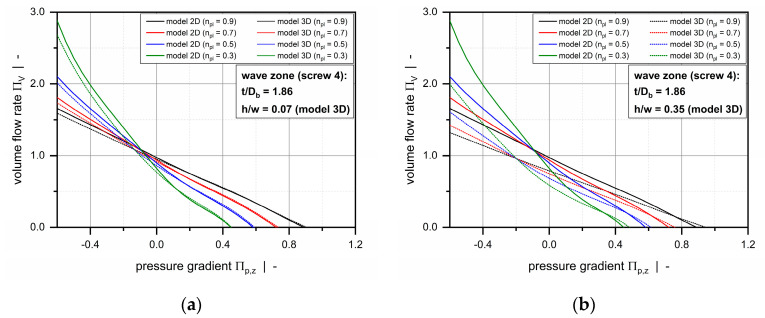
Comparison between the two- and three-dimensional melt-conveying models: Screw characteristic curves for the wave zone of screw 4 with t/D_b_ = 1.86, showing the pumping capability of a wave peak (**a**) and valley (**b**).

The two-dimensional melt-conveying model has three independent dimensionless input parameters: (i) the screw pitch ratio $t/D_{b}$, (ii) the power-law index n, and (iii) a dimensionless pressure gradient $\Pi_{p,z}$. Taking the influence of the screw flights into account, the three-dimensional model additionally requires (iv) the aspect ratio of the screw channel *h/w* to be predefined. A comparison of the models is shown in Figure 6, which illustrates two- and three-dimensional screw characteristic curves for the wave zone of screw 4 (with *t/D_b_ =* 1.86) and various power-law indices. To illustrate the effect of the screw flights on the flow, the aspect ratio in the three-dimensional model was set to *h/w* = 0.07 and *h/w* = 0.35, representing the screw channels at a peak and a valley, respectively.

Analyzing the conveying behavior at the wave peak (Figure 6a), the two- and three-dimensional screw characteristic curves show a similar behavior due to the limited influence of the screw flights. In contrast, a significant difference is evident at the wave valley (Figure 6b), where the screw flights reduce the flow rate for a variety of pressure gradients. The impact of the screw flights is more pronounced for negative dimensionless pressure gradients as found in overridden melt-conveying zones. A drawback of the three-dimensional model is that the screw flights are represented as being equally large on both sides of the channel. In wave-dispersion screws, the barrier flight is undercut relative to the main flight. This effect can be of significant size in the case of energy-transfer screws, in which the functions of the main and the secondary flight alternate along the functional zone. To consider the different clearances of the active and passive flights and their diverse impacts on the flow, the following correction factor is introduced:

(5) $$f^{*}=\frac{2h-\delta_{f}-\delta_{b}}{2h}$$

where $\delta_{f}$ and $\delta_{b}$ are the clearances of the main and the barrier flight, respectively. The correction factor relates the channel depth covered by the flights to the total possible channel depth of 2 h. In the following screw calculation routine, it is used to weight the impacts of the two- and three-dimensional melt-conveying models in the output prediction:

(6) $$\Pi_{V}=f^{*}\Pi_{V,3d}+\left(1-f^{*}\right)\Pi_{V,2d}$$

### 3.2. Network Analysis

In Part A [34], we developed a calculation routine based on network theory to analyze pressure-driven flows in double-wave zones. Omitting the influence of the drag flow, the theory predicts the pressure distribution, the local down- and cross-channel flows, and the transverse mixing capability. This section revisits the fundamentals of the modeling approach and highlights the novel features incorporated to include the drag force of the screw. The usefulness of network theory in the flow analysis of extrusion equipment has been demonstrated by several studies [5,35,36,37,38,39,40].

**Figure 7:**
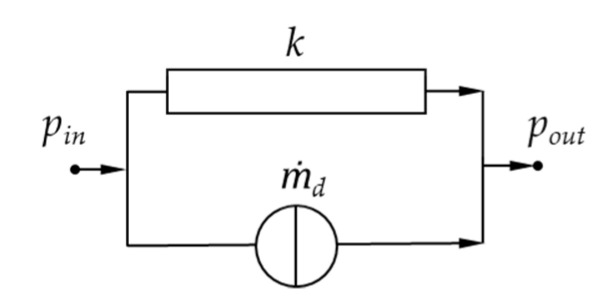
Basic network element for melt conveying.

The main idea of our semi-numerical approach is as follows: To simplify the complex flow in wave-dispersion zones, the screw channel is subdivided into very small segments with constant geometrical and physical parameters. These sections are indicated by network elements, each of which consists of a source and a resistance connected in parallel that represent the local drag and pressure flows, respectively. With these properties, the network elements are designed to describe the local output-pressure gradient relationship. Figure 7 illustrates the basic structure of a network element, where ${\dot{m}}_{d}$ is the drag flow, *k* the conductance, and $p_{in\;}$ and $p_{out}$ are the pressures at the surrounding nodes.

The mass flow rate of an element is given by a linear combination of the drag and pressure flow. The latter is defined by the conductance and the pressure difference between the nodes:

(7) $$\dot{m}={\dot{m}}_{d}+{\dot{m}}_{p}={\dot{m}}_{d}+k\left(p_{in}-p_{out}\right)$$

**Figure 8:**
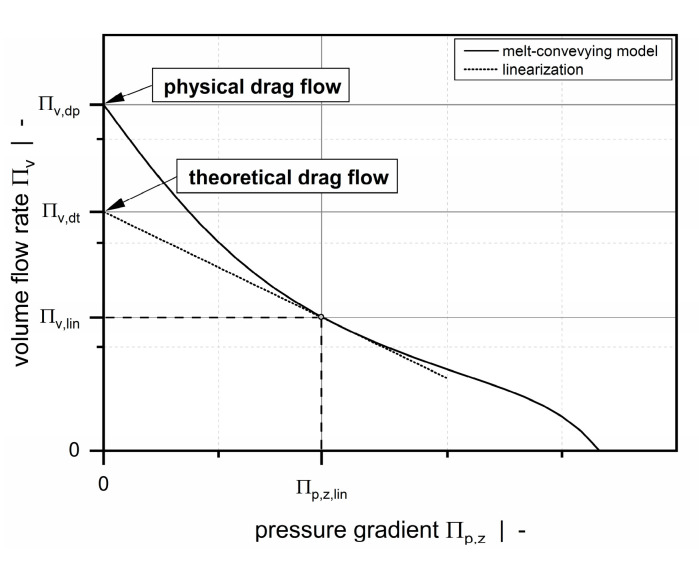
Linearization of the nonlinear screw characteristic curve at operating point $\left(\Pi_{p,z}|\Pi_{V}\right)$.

In Part A [34], we linearized a nonlinear die-flow equation for power-law fluids to determine the properties (theoretical drag flow and conductance) of each network element. The modeling approach presented here considers the nonlinear conveying behavior of the wave channel by linearizing our melt-conveying models $\Pi_{V}=f\left(t/D_{b},\;n,{\;\Pi }_{p,z}\right)$ and $\Pi_{V}=f\left(h/w,\;t/D_{b},\;n,{\;\Pi }_{p,z}\right)$ with respect to the current operating point. For both models, (theoretical) drag flow and conductance are obtained from the initial value and the slope of the linearization, respectively (Figure 8).

The final element properties are then evaluated by weighting the results according to our correction factor in Equation (5). Note that the theoretical drag flow used in the network calculation deviates from the physical drag flow.

**Figure 9:**
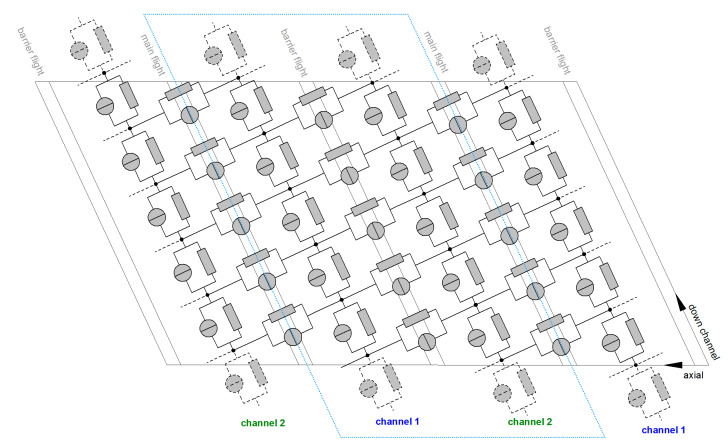
Network discretization of an unwound double-wave zone consisting of (i) two subchannels, (ii) two main flights, and (iii) a barrier flight. The network elements in the down- and cross-channel directions are connected via nodal points.

Analogously to electrical circuits, the network elements are connected via nodal points. The axial and down-channel positions of the nodal points result from the screw geometry and a predefined discretization distance. Figure 9 illustrates the flow network of an unwound double-wave zone consisting of two subchannels (channels 1 and 2), two main flights, and a barrier flight. We used down-channel elements to describe the flow along the wave channels and cross-channel elements to include transverse flow. Compared to the theory presented in Part A [34], the flow network was extended to consider both transverse mixing over the barrier flight and leakage flow over the main flight.

As in the previous part [34], the cross-channel network elements were initialized with three elements connected in series: (i) one element from the channel center to the pushing flight, (ii) one element over the flight, and (iii) one element from the trailing flight to the center of the channel one turn behind. This procedure allowed us to accurately model the stepwise changes in channel height between the subchannels. The elements were then replaced by an equivalent element. To identify the pairs of nodal points connected in the cross-channel direction, we introduced a parameter counting the number of down-channel elements between these nodes:

(8) $$N_{off}=round\left(\;\frac{\frac{D_{b}\;\pi }{cos\left(\varphi_{b}\right)}-\left(w_{1}+w_{2}+e_{a}+e_{b}\right)\;tan\left(\varphi_{b}\right)}{\frac{D_{b}\;\pi }{N_{z}\;cos\left(\varphi_{b}\right)}}\right)$$

where $w_{1}$, $w_{2}$, $e_{a}$, and $e_{b}$ are the widths of the first subchannel, the second subchannel, the main flight, and the barrier flight, respectively. The parameter additionally depends on the resolution of the network determined by the number of elements per revolution $N_{z}$. In contrast to the down-channel flow, transverse flow is described by a Newtonian output-pressure-gradient relationship based on representative viscosities. The flow in the cross-channel direction is in fact two-dimensional since the rate-limiting influence of the screw flight can be ignored. Note that our two-dimensional melt-conveying model is only valid if $0.6<t/D_{b}<2.4$ or $10.8{}^{\circ}<\varphi_{b}<37.4{}^{\circ}$ [30]. In the transverse direction with $90{}^{\circ}-\varphi_{b}$, these conditions are not fulfilled as the cross-channel component dominates the flow. In order to consider the specific flow conditions over the screw flight, new melt-conveying models for shear-thinning polymer melts are currently under development.

**Figure 10:**
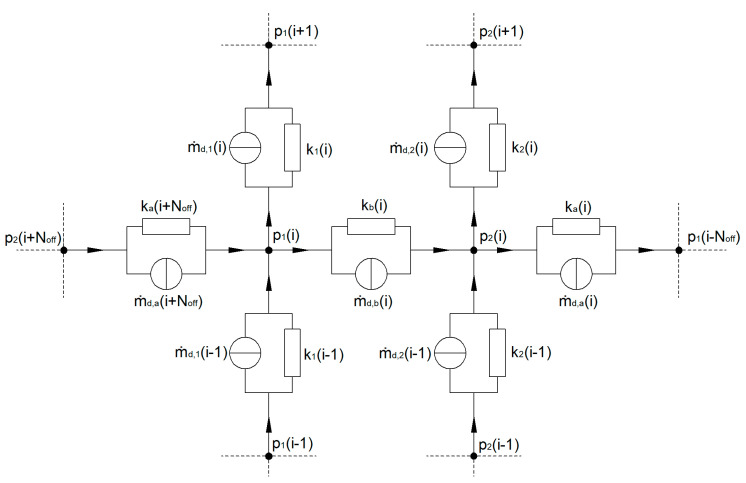
Equivalent circuit diagram.

The equivalent circuit diagram for an arbitrary nodal point with index *i* is shown in Figure 10. The indices “1” and “2” describe the first and the second subchannel, while the indices “a” and “b” refer to the main and the barrier flight.

For solving the flow network, nodal analysis is applied. For each non-reference node the sum of the incoming flows must equal the sum of outgoing flows (cf. Kirchhoff’s current law):

(9) $$\underset{i}{\sum }{\dot{m}}_{i}=0$$

The network equations for an arbitrary nodal point with index i are given by:

Channel 1:

(10) $$\begin{array}{cc} k_{1}(i-1)\;p_{1}(i-1) & +\left[-k_{1}\left(i-1\right)-k_{1}\left(i\right)-k_{b}\left(i\right)-k_{a}\left(i+N_{off}\right)\right]\;p_{1}\left(i\right)+k_{b}\left(i\right)\;p_{2}\left(i\right)\\ & +k_{1}\left(i\right)\;p_{1}\left(i+1\right)+k_{a}\left(i+N_{off}\right)\;p_{2}\left(i+N_{off}\right)\\ & =-{\dot{m}}_{d,1}\left(i-1\right)+{\dot{m}}_{d,1}\left(i\right)+{\dot{m}}_{d,b}\left(i\right)-{\dot{m}}_{d,a}\left(i+N_{off}\right) \end{array}$$

Channel 2:

(11) $$\begin{array}{cc} k_{a}\left(i\right)\;p_{1}(i-N_{off}) & +k_{2}\left(i-1\right)\;p_{2}\left(i-1\right)+k_{b}\left(i\right)\;p_{1}\left(i\right)\\ & +\left[-k_{2}\left(i-1\right)-k_{b}\left(i\right)-k_{2}\left(i\right)-k_{a}\left(i\right)\right]\;p_{2}\left(i\right)+k_{2}\left(i\right)\;p_{2}\left(i+1\right)\\ & =-{\dot{m}}_{d,2}\left(i-1\right)-{\dot{m}}_{d,b}\left(i\right)+{\dot{m}}_{d,2}\left(i\right)+{\dot{m}}_{d,a}\left(i\right) \end{array}$$

Taking all nodes of the flow network into account yields a system of linear equations that can be summarized in matrix form:

(12) $${\dot{m}}_{d}+K\cdot p=\dot{m}$$

where ${\dot{m}}_{d}$ is the drag flow vector, K the conductance matrix, and p the pressure vector, and $\dot{m}$ includes the boundary conditions. The solution of this equation system yields the nodal pressures:

(13) $$p=K^{-1}\cdot \left(\dot{m}-{\dot{m}}_{d}\right)$$

Particular attention must be paid to the network equations for the first couple of nodes in each subchannel. For i=1 the inlet mass flow rates in both channels ${\dot{m}}_{1,0}$ and ${\dot{m}}_{2,0}$ are known, while for $i<N_{off}$ there are no cross-channel connections over the main flight to channel 1. In addition, the equations must be modified for the last several nodes. For i=n+1 the outlet pressures of both channels are given by $p_{1}\left(n+1\right)=p_{1,out}$ and $p_{2}\left(n+1\right)=p_{2,out}$, while for $i<n+1-N_{off}$ there are no cross-channel connections over the main flight to channel 2. The inlet mass-flow rates are given by:

(14) $${\dot{m}}_{1,0}=\;\frac{{\dot{m}}_{0}{\;w}_{1}\left(1\right)}{w_{1}\left(1\right)+w_{2}\left(1\right)}\;\;\;\;\;\;\;\;\text{and}\;\;\;\;\;\;\;\;{\dot{m}}_{2,0}=\;\frac{{\overset{\text{˙}}{m}}_{0}{\;w}_{2}\left(1\right)}{w_{1}\left(1\right)+w_{2}\left(1\right)}$$

where ${\dot{m}}_{0}$ is the output of the single-screw extruder.

### 3.3. Screw Simulation

**Figure 11:**
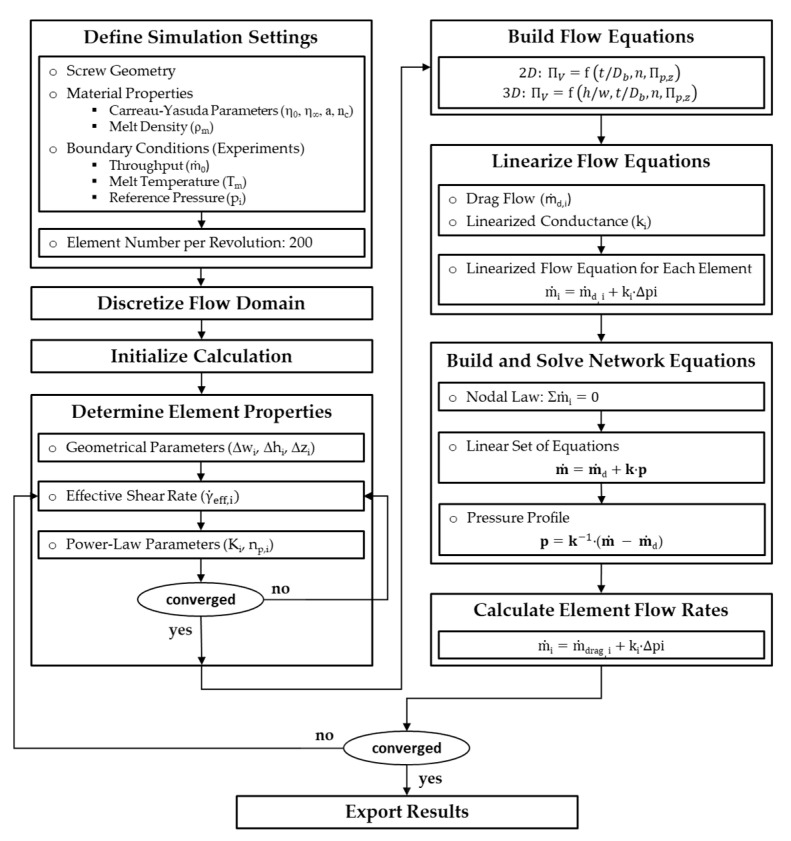
Flow chart of our semi-numerical modeling approach based on network theory.

Our semi-numerical simulation routine is based on the procedure shown in Figure 11. A detailed discussion was given in Part A [34].

At the beginning, the main simulation settings were configured. These include: (i) input of screw geometry, material properties, and processing conditions (screw speed, mass flow rate, melt temperature, and reference pressure), (ii) selection of material models, and (iii) definition of parameters required for the solving process. Using the data obtained from the experimental tests, the inlet mass flow rates were defined according to Equation (14). For each wave section, the last experimental pressure value ($p_{4}$ or $p_{5}$) was used to define a reference pressure along the wave zone. Our simulations considered an isothermal flow of an incompressible polymer melt. For this reason, the melt temperature was used to shift the viscosity data to the desired temperature level, while the density was constant. For all calculations, the number of network elements per revolution was set to $N_{z}=200$.

In the next step, the screw channel was discretized into a network of small segments, and the calculation was initialized. The iteration scheme started by determining the properties of the network elements. At each nodal point, the geometrical parameters were calculated and assigned to the connected network elements. To evaluate the rheological element properties, the Carreau-Yasuda parameters were converted into equivalent power-law parameters for the local shear rate. For detailed information, the reader is referred to Part A [34].

In the subsequent step, linearized element properties were derived (${\dot{m}}_{d}$ and k), and the network equations for each element were built. The resulting system of linear equations was then solved, and the calculated pressure field was used to update the element flow rates for the next iteration. A simulation was considered converged if the pressure differences between the first and final nodes in each subchannel was smaller than ∆p<0.01 bar between two iteration loops.

With the simulation procedure having converged, the modeling procedure provided (i) axial pressure profiles along the subchannels and (ii) mass flow rates in each subchannel and over the screw flights.

## 4. Results and Discussion

### 4.1. Comparison of Experimental and Calculated Results

The semi-numerical modeling approach presented in this article was used to reproduce the pressure profiles measured in the experimental part. For each operating point, we predefined the screw speed, the output, and the melt temperature according to our experimental results and calculated the pressure profiles along the subchannels of the wave zone.

For convenience, these results were averaged to obtain mean pressure curves for the double wave-channel system. Pressures were evaluated over the entire length of the wave section, except for screw 1, where the wave zone started at an axial position of 6.4 D_b_. Since no experimental pressures were measured in the early sections of the screw, we started the calculation at 12.5 D_b_.

**Figure 12:**
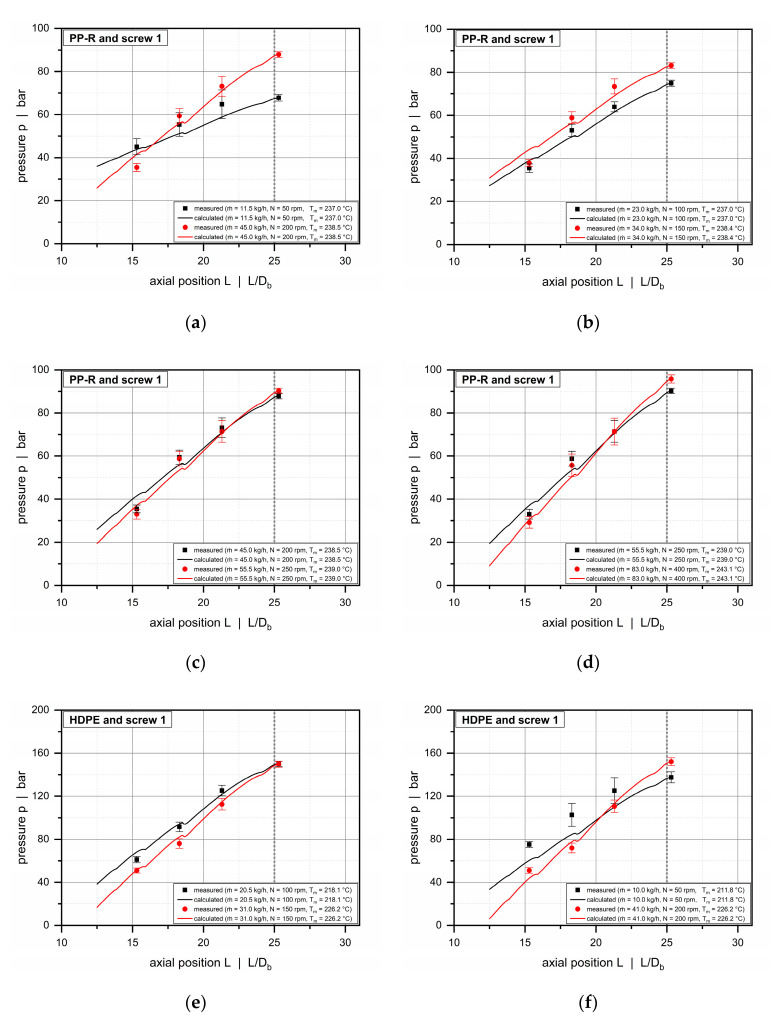
Comparison between experimental data and calculated solutions for barrel 1 and screw 1. Axial pressure profiles along the wave zone for PP-R (**a**–**d**) and HDPE (**e**,**f**). The continuous lines indicate the solutions of our semi-numerical modeling approach, while the circles illustrate experimental data.

**Figure 13:**
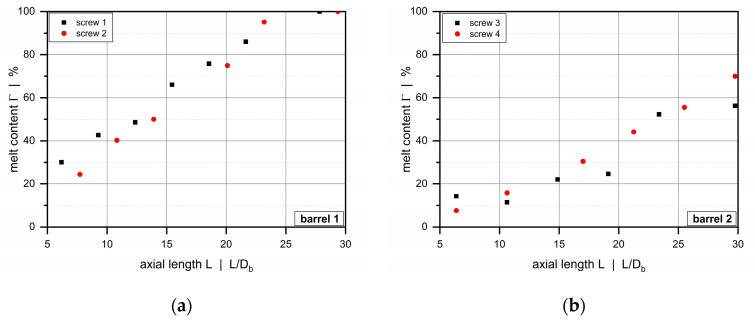
Melting profiles for HDPE for the smooth-bore extruder (barrel 1) (**a**) and the helically grooved-feed extruder (barrel 2) (**b**).

Figure 12 shows axial pressure profiles for barrel 1 (smooth-bore extruder) and screw 1 for various operating conditions. The calculated pressure profiles are in excellent agreement with the measured data, taking the averaging of the calculated results and the standard deviation of the experimental data into account. Especially in the early screw sections, this result seems surprising, as the melt-conveying models applied in our semi-numerical modeling approach assume the polymeric material to be entirely in a liquid state, and the influence of solid resin particles on the conveying characteristics is ignored. The melting profiles in Figure 13 clearly indicate that for the screw-barrel system under investigation (barrel 1 and screw 1) the melt content is in the range of 50% at the beginning of the wave zone and increases steadily over the length of the functional zone.

**Figure 14:**
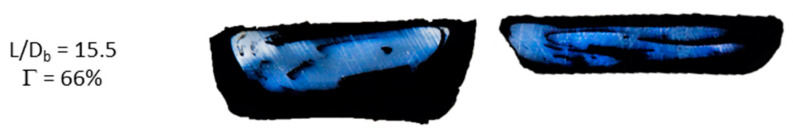
Cross section of polymer ribbon of both channels extracted at an axial position of 15.5 L/D_b_ during a solidification experiment with barrel 1 and screw 1. The white and black sections indicate solids and polymer melt, respectively.

**Figure A1:**
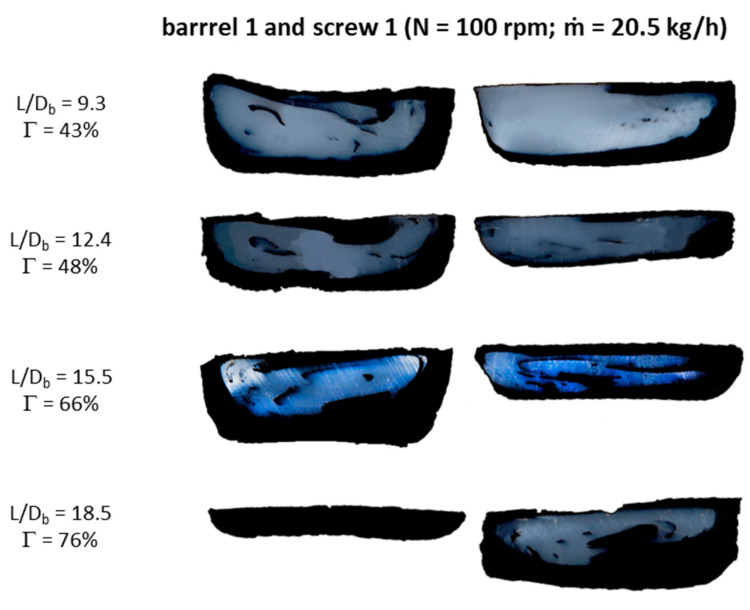

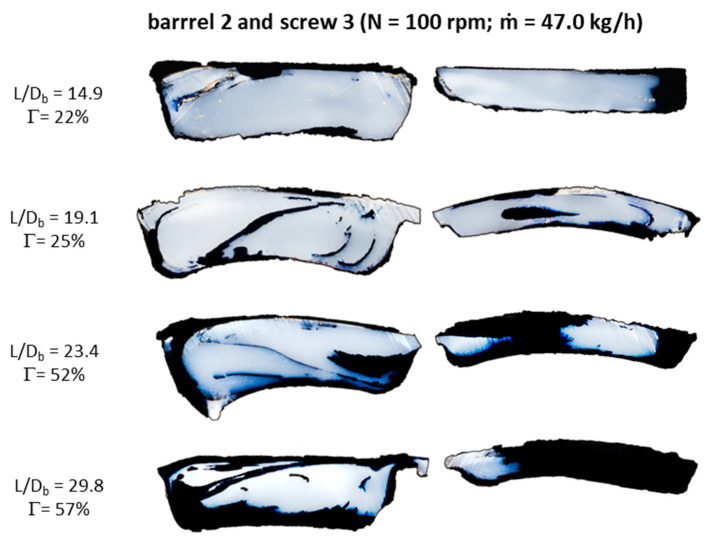
Cross sections along the wave zone obtained from the solidification experiments for two screw-barrel configurations: barrel 1 and screw 1, and barrel 2 and screw 3. Both tests were carried out at a screw speed of N=100 rpm, which resulted in a throughput of $\dot{m}=20.5$ kg/h and $\dot{m}=47.0$ kg/h for the first and the second screw-barrel configuration, respectively.

The high accuracy of the melt-conveying models in the early screw sections may be a result of the specific way in which wave-dispersion screws melt dispersed solids, which gives rise to particle clusters and individual particles trapped within the polymer melt (Figure 14). Since melt films are generated on each side of the screw channels, the viscous-drag conveying behavior replaces frictional-drag conveying behavior at an early stage. This change is an important prerequisite for the validity of our melt-conveying models. The melt films are subjected to high shear rates, thus greatly affecting the viscosity and the pressure behavior of the system. The solid bulk material in the channel centers, in contrast, shows minor viscosity differences and has therefore less influence on the pressure characteristics. This behavior is also represented by our melt-conveying models. In a fully melt-dominant description, however, the effect is explained not by the presence of solids, but by the reduced shear rates at the channel centers (especially in overridden functional zones), which cause the viscosities to reach the zero-viscosity plateau. The cross sections obtained from our solidification experiments are shown in Figure A1 in the Appendix A. For the smooth-bore system, melting completed after roughly 25 D_b_, whereas the helically grooved barrel showed a melt content of approximately 70% at the end of the wave zone. In the latter case, the shearing element converted the remaining solids.

Characteristic of smooth-bore extruders, the wave-dispersion zones analyzed in Figure 12 generate pressure for all setups, as indicated by a positive pressure gradient. With increasing screw speed, the slope of the pressure profiles, and hence the pressure build-up capacity, increases. The latter is affected by the geometry of the wave channels and the cross-channel flows induced by the drag force of the screw and the transverse pressure gradients.

**Figure 15:**
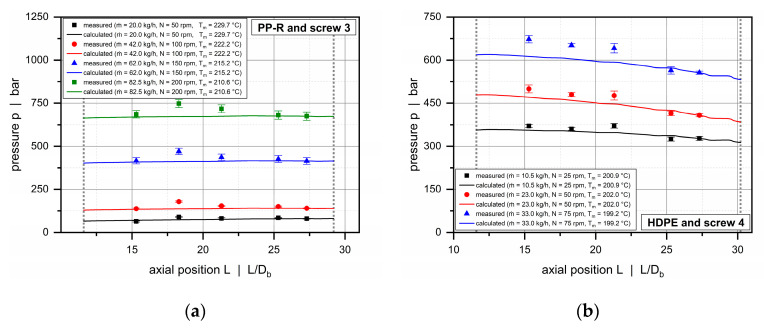
Comparison between experimental and calculated data for barrel 2. Axial pressure profiles along the wave zone for PP-R and screw 3 (**a**) and HDPE and screw 4 (**b**).

**Figure 16:**
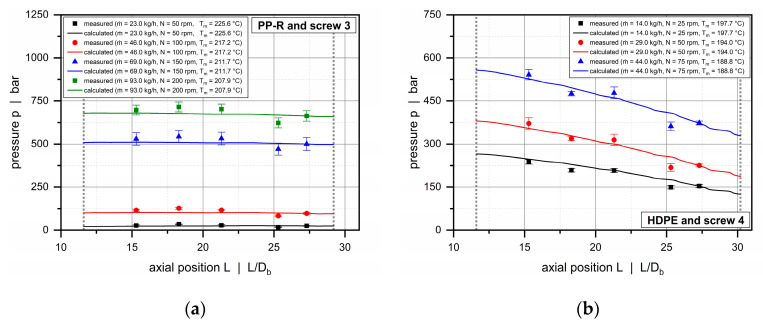
Comparison between experimental and calculated data for barrel 3. Axial pressure profiles along the wave zone for PP-R and screw 3 (**a**) and HDPE and screw 4 (**b**).

Compared to the conventional extruder (barrel 1), the double-wave screws used in combination with the helically grooved and the axially grooved barrels (barrels 2 and 3) operate balanced or overridden with respect to the governing pressure characteristics. For these systems, the extrusion process is feed-controlled, and pressure development occurs early in the solids-conveying zone, while the downstream functional zones work pressure-neutral or consume pressure. Figure 15 and Figure 16 illustrate axial pressure profiles for barrels 2 and 3, respectively. For both configurations, pressure profiles are shown for screws 3 and 4 in which the wave sections extend from 11.6 D_b_ to nearly the end of the screws. Similarly, the diagrams compare the solutions of our semi-numerical modeling approach and the experimental results ($p_{1}$ to $p_{5}$) for various setups.

As expected, by constructing the barrels with grooves, the output was more than doubled from barrel 1 to 2, and again significantly raised from barrel 2 to 3. In addition, a substantial increase in pressure from 200 to 800 bar is evident. Our main intention in using different barrels was to allow the processing unit to operate at various specific output rates to increase the process windows for model validation. For all setups, the calculated pressures accurately reflect the experimental pressure characteristics. The less viscous PP-R shows nearly constant pressures over the entire length of the wave zone, whereas the more viscous HDPE is subjected to a negative pressure gradient in both barrel systems.

For all experimental setups, pressure fluctuations caused by the presence of solid resin particles were within an acceptable range, as shown by the standard deviations obtained for the experimental data.

Using our two- and three-dimensional melt-conveying models in combination with network theory enables fast prediction of the conveying characteristics of double-wave screws. The modeling approach being based on a fully melt-dominant description, the method applies several modeling assumptions that can further be optimized. To increase the accuracy of the approach, the presence of solid material and phase transition effects can be taken into account. The extrusion literature provides several solids-conveying and melting models that may be implemented into the network calculation. A full description of the transport phenomena, however, was not the objective of this research.

The presented simulation routine uses two- and three-dimensional melt-conveying models to analyze local conveying characteristics of the wave zone. These models consider flows of shear-thinning polymer melts in unwound screw channels and ignore the influence of channel curvature. While the flat-plate assumption is widely used in extrusion theory and has often proven useful in screw analyses, the applicability for wave-dispersion zones remains questionable. Especially in the deep valley portions of the wave zones, the channel curvature reaches a critical level with the ratio of channel depth to screw diameter significantly exceeding the values typically found in conventional metering zones. In a recent article [41], we showed that for deep screw channels the flat-plate model underestimates the flow rate for a variety of processing conditions.

Another simplification made in the development of the melt-conveying models is the assumption of the flow as isothermal. Due to the temperature-dependent viscosity of the polymer melts, the velocity and temperature fields in the screw channel are in fact coupled. Roland et al. [40] presented an extended modeling approach based on network theory to predicting the non-isothermal conveying characteristics of conventional metering zones.

### 4.2. Distribution of Mass-Flow Rates and Pressure

**Figure 17:**
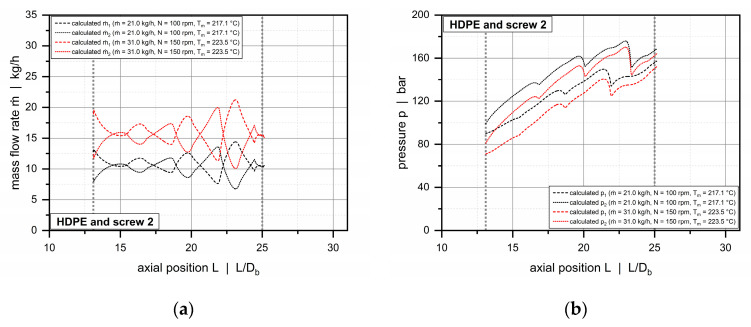
Distribution of mass-flow rates (**a**) and pressures (**b**) in subchannels 1 and 2 for barrel 1 (smooth-bore extruder) and screw 2. The dashed and dotted lines represent the solutions for subchannels 1 and 2, respectively.

**Figure 18:**
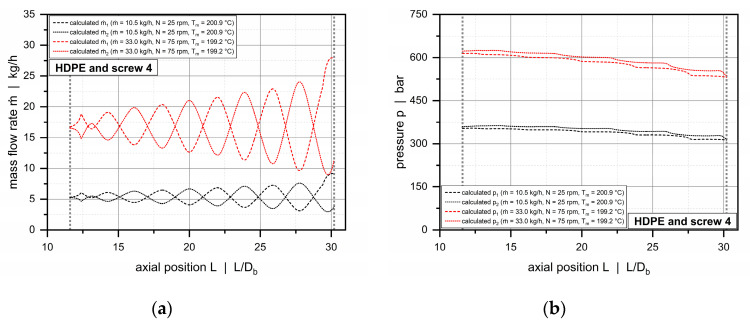
Distribution of mass-flow rates (**a**) and pressures (**b**) in subchannels 1 and 2 for barrel 2 (helically grooved extruder) and screw 4. The dashed and dotted lines represent the solutions for subchannels 1 and 2, respectively.

This section presents the results of applying our network-based simulation routine in order to analyze the mass flow rates and pressures in the subchannels of the double-wave zones. Figure 17 and Figure 18 illustrate the behavior for a conventional (barrel 1 and screw 2) and a feed-controlled extrusion process (barrel 2 and screw 4).

The distribution of the mass-flow rates is governed primarily by the oscillating channel-depth profiles of the wave channels. Material flowing down a channel toward a peak is forced to split flow due the diminishing cross-sectional area. Some material portions remain in the original channel, and others travel across the barrier flight to increase the mass flow rate in the adjacent channel. This process is repeated multiple times throughout the wave-dispersion zone.

Taking the channel-depth profiles in Figure 3 into account, the local mass flow rates reach a maximum when the channel under investigation decreases in cross-sectional area, while the adjacent channel increases in cross-sectional area. Depending on the ascending or descending nature of the channel depth, extreme values are found in the region where the cross-sectional areas in the subchannels are equally large. The exact position depends on the interaction of the flow channels, the transverse pressure gradient and the drag force of the screw. Once this position has been reached, the direction of cross-channel flow changes.

For both extrusion processes, the pressures in channel 2 are generally higher than in channel 1. This is caused by the different clearances of the main and the barrier flights. At the pushing side of channel 2, transverse flow is largely restricted by the small clearance of the main flight. In contrast, the increased clearance of the barrier flight allows the material to transfer between the channels. For a majority of operating conditions, the standard deviations of the pressures evaluated in the experimental part exceeded slightly the calculated pressure differences between the subchannels.

Due to compression of the wave zones in the axial direction, cross-channel mixing improves with increasing screw length, as indicated by the rising amplitudes in the mass-flow distributions. To evaluate the transverse mixing performance, the following mixing index is introduced:

(15) $$\kappa =\frac{\sum_{i}\left|{\dot{m}}_{f,i}\right|}{{\dot{m}}_{0}}$$

where the numerator represents the sum of the cross-channel element flow rates (over the main and the barrier flights) and the denominator the total output. The higher the mixing parameter, the more pronounced the transverse flow and thus cross-channel mixing.

**Figure 19:**
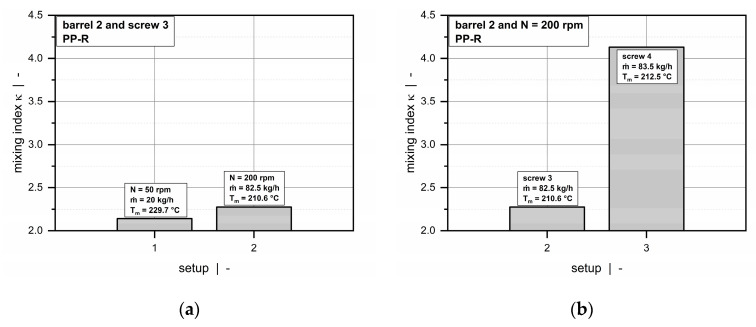
Comparison of mixing indices for various processing conditions: Influence of screw speed for a given screw-barrel-material configuration (**a**) and influence of screw design for a given operating point (**b**).

Figure 19 compares the influence of screw speed and screw design on the transverse mixing performance. For a given experimental setup (screw-barrel-material combination), cross-channel mixing improves only slightly with increasing screw speed. As the sum of cross-channel flow rates and the total throughput increase nearly proportionally if the screw speed is raised (setups 1 and 2), a similar mixing index is obtained in both cases, indicating that the total throughput passes across the screw flights more than twice along the wave zone. In contrast, a significant difference in mixing performance is evident if the screw design is modified. For a given operating point, the mixing index is almost doubled if the number of peaks and valleys along the wave zone is increased (setups 2 and 3). Further comparisons were carried out to investigate the influence of the viscosity behavior. Again, we observed no significant differences in transverse mixing between the lower and the higher viscous material.

## 5. Conclusions

We have presented and validated a semi-numerical modeling approach to analyzing flows of shear-thinning polymer melts in flow systems with alternating wave channels. In the first part (Part A), we developed the mathematical groundwork for the theory presented here by focusing exclusively on pressure-driven flows. Reducing the complexity of the mathematical analysis in Part A allowed us to propose a network-based modeling approach to predicting: (i) the pressure distribution, (ii) the local down- and cross-channel flows, and (iii) the transverse mixing capability of double wave-channel systems. Rather than solving the full set of conservation equations, the approach iteratively solves a system of linear equations. For a number of experimental setups, the semi-numerical modeling approach was shown to yield accurate results at low computational cost.

While the method proposed in Part A can be used to model flows in various types of extrusion dies with changing channel geometry, the research presented here incorporated the influence of the screw rotation into the network-based calculation. Two significant modifications were made to extend the original theory.

First, to describe the local conveying characteristics of the wave zone, two- and three-dimensional melt-conveying models for shear-thinning polymer melts were combined, using a novel correction factor. For each subchannel, the parameter relates the distance covered by the flight flanks on both sides of the channel to the total possible channel depth, thereby providing a measure for determining the impact of the screw flights in the output calculation. In wave-dispersion screws, the barrier flight is typically undercut with respect to the main flight to allow the material to transfer between the subchannels. Assuming the polymer melt to stick to the walls of the screw channel, the rate-limiting influence of the secondary flight is therefore reduced compared to that of the main flight.

Second, we adapted the structure of the flow network to include both transverse flow over the barrier flight and leakage flow over the main flight. A Newtonian output-pressure-gradient relationship based on representative viscosities was used to describe transverse flow. The cross-channel elements in the flow network being positioned perpendicular to the screw channel, new melt-conveying models for shear-thinning polymer melts are currently under development that consider the specific flow conditions over the screw flights.

The validity of the network-based modeling approach was confirmed experimentally for both conventional and feed-controlled extrusion processes by analyzing a number of double-wave screws and processing conditions. Our main intention in using extruder barrels of different designs was to increase the parameter space available for model validation. The results of our semi-numerical modeling approach for the axial pressure profiles along the wave zones accurately reproduced the experimental data. Even in the screw sections with increased quantities of solid particles, our melt-dominant analysis provided highly satisfactory results.

Our network-theory-based simulation routine enables fast prediction of the conveying behavior of double-wave screws. Taking the influence of the shear-thinning flow behavior of polymer melts into account, the modeling approach permits a simple description of the local flow phenomena by a linear superposition of a drag and a pressure flow. Due to its efficient solving process, the modeling approach is expected to be particularly useful in (i) design and optimization studies and (ii) process troubleshooting of wave-dispersion screws. In both cases, a fast and accurate analysis of the screw concept is crucial.

## Figures and Tables

**Table 1:** Material parameters ($\rho_{m}$ is the melt density at 200 °C and 200 bar).

Parameter	Unit	HDPE	PP-R
$\eta_{0}$	Pas	95,230	2957
$\eta_{\infty }$	Pas	0	0
λ	s	3256	0.173
$n_{c}$	-	0.289	0.425
a	-	1	1
α	1/K	0.0138	0.0162
$T_{0}$	K	473.15	473.15
$\rho_{m}$	kg/m^3^	752	730

**Table 2:** Temperature profiles in °C.

Material	$T_{1}$	$T_{2}$	$T_{3}$	$T_{4}$	$T_{5}$
HDPE	40	190	200	200	200
PP-R	40	230	240	240	240

**Table 3:** Comparison of barrel designs.

Number	Barrel	Groove Design				
		Number	Width	Height	Length *	Pitch
Barrel 1	Smooth	-	-	-	-	
Barrel 2	Helically grooved	6	5 mm	2.8 mm	140 mm	105 mm
Barrel 3	Axially grooved	6	8 mm	2.8 mm	140 mm	∞

* measured from the front end of the hopper.

**Table 4:** Comparison of double-wave zones.

Dimensions		Unit	Screw 1	Screw 2	Screw 3	Screw 4
Outer diameter	*D_S_*	mm	34.85	34.85	34.85	34.85
Axial start of wave zone *	*L*_1_	mm	225	459.5	407	407
Axial end of wave zone *	*L*_2_	mm	876.5	880.0	1021.5	1056.5
Length of wave zone	ΔL	mm	651.5	420.5	614.5	649.5
Pitch	*t*	mm	45.5	49.0	65.0	65.0
Pitch angle	$\varphi_{b}$	°	22.4	24.0	30.6	30.6
Flight width	*e*	mm	3.2	3.5	3.5	3.5
Number of parallel flights	*i*	-	2	2	2	2
Barrier undercut	$\delta_{b}$	mm	0.6	0.6	1.45	1.45
Flight clearance	$\delta_{f}$	mm	0.075	0.075	0.075	0.075

* measured from the front end of the hopper.

**Table 5:** Positions, measuring ranges, and response times of pressure transducers.

Pressure Sensor	Axial Position	Range	Response Time
	L/D_b_	bar	ms
$p_{1}$	15.3	0–1000	8
$p_{2}$	18.3	0–1000	8
$p_{3}$	21.3	0–1000	8
$p_{4}$	25.3	0–1000	8
$p_{5}$	27.3	0–1000	8
$p_{6}$	34.7	0–500	8

## Appendix A

**Table A1:** Operating points. Mean values of process parameters measured in the experimental part.

Test	Barrel	Screw	Material	N	$\dot{m}$	$T_{M}\;$	$p_{1}\;$	$p_{2}\;$	$p_{3}\;$	$p_{4}\;$	$p_{5}\;$	$p_{6}\;$
-	-	-	-	rpm	kg/h	°C	bar	bar	bar	bar	bar	bar
1	1	1	HDPE	50	10.0	211.8	75.2	102.6	125.2	137.5	-	184.2
2	1	1	HDPE	100	20.5	218.1	61.2	91.5	125.3	150.1	-	213.4
3	1	1	HDPE	150	31.0	222.6	50.9	76.1	112.6	149.7	-	232.2
4	1	1	HDPE	200	41.0	226.2	51.0	71.9	110.7	152.1	-	245.8
5	1	1	HDPE	250	49.0	230.6	56.0	73.8	155.0	158.1	-	270.1
6	1	1	HDPE	300	58.0	234.4	62.1	81.6	122.1	165	-	290.5
7	1	1	HDPE	350	67.0	239.3	65.5	82.7	122.0	170.6	-	292.5
8	1	1	HDPE	400	76.5	249.7	65.5	85.5	128.1	180.6	-	297.4
9	1	1	PP-R	50	11.5	237.0	45.1	55.4	64.8	67.8	-	85.7
10	1	1	PP-R	100	23.0	237.0	35.4	53.0	64.0	74.9	-	103.9
11	1	1	PP-R	150	34.0	238.4	37.9	58.9	73.4	83.1	-	114.5
12	1	1	PP-R	200	45.5	238.5	35.4	59.4	73.1	87.9	-	123.5
13	1	1	PP-R	250	55.5	239.0	32.9	58.7	71.4	90.2	-	129.7
14	1	1	PP-R	300	65.0	238.4	24.2	50.5	67.0	89.5	-	136.6
15	1	1	PP-R	350	74.5	239.4	28.2	54.4	96.9	92.9	-	140.8
16	1	1	PP-R	400	83.0	243.1	29.1	55.7	71.4	95.8	-	148.1
17	1	2	HDPE	50	11.5	208.0	122.1	158.5	158.0	173.9	-	185.7
18	1	2	HDPE	100	21.0	217.1	77.0	126.3	130.2	163.2	-	209.4
19	1	2	HDPE	150	31.0	223.5	89.4	124.5	129.4	158.5	-	227.9
20	1	2	HDPE	200	41.0	227.2	103.2	129.4	134.2	156.0	-	243.9
21	1	2	HDPE	250	48.0	233.3	122.8	140.4	117.4	145.7	-	253.7
22	1	2	HDPE	300	58.0	238.3	135.8	153.0	126.7	153.5	-	265.8
23	1	2	HDPE	350	67.5	244.1	149.6	167.3	136.0	161.7	-	277.8
24	1	2	HDPE	400	77.0	250.1	163.1	180.5	143.2	172.7	-	288.9
25	1	2	PP-R	50	12.5	238.8	73.0	97.3	81.3	98.6	-	104.9
26	1	2	PP-R	100	22.5	239.1	52.0	90.1	81.2	105.3	-	123.6
27	1	2	PP-R	150	33.0	242.2	51.4	91.4	83.3	108.2	-	135.0
28	1	2	PP-R	200	44.0	240.7	36.6	89.3	80.0	110.1	-	144.5
29	1	2	PP-R	250	54.5	240.4	37.3	105.4	89.8	120.3	-	154.9
30	1	2	PP-R	300	64.5	238.6	45.0	107.1	99.7	125.9	-	164.0
31	1	2	PP-R	350	76.0	240.2	48.2	123.1	106.7	133.0	-	170.7
32	1	2	PP-R	400	87.0	240.9	68.4	127.6	111.7	136.2	-	174.1
33	2	3	HDPE	25	11.0	196.6	109.6	117.5	114.5	102.7	97.5	182.8
34	2	3	HDPE	50	23.0	199.4	137.8	157.7	142.9	125.3	119.3	192.4
35	2	3	HDPE	75	36.0	192.1	220.1	248.8	222.2	205.0	201.0	227.6
36	2	3	HDPE	100	47.0	181.7	356.4	392.5	359.0	335.7	335.0	294.5
37	2	3	HDPE	125	59.5	175.6	453.4	495.0	459.7	429.8	434.0	328.7
38	2	3	HDPE	150	72.0	171.5	538.4	585.9	546.1	508.2	519.2	340.6
39	2	3	PP-R	50	20.0	229.7	64.8	90.2	81.5	85.1	80.3	113.9
40	2	3	PP-R	100	42.0	222.2	138.3	179.3	154.5	150.1	140.2	144.9
41	2	3	PP-R	150	62.0	215.2	415.9	470.7	436.7	426.2	414.4	326.6
42	2	3	PP-R	200	82.5	210.6	685.8	747.7	717.7	681.1	674.5	280.0
43	2	4	HDPE	25	10.5	200.9	370.8	360.8	371.6	325.4	327.7	7.8
44	2	4	HDPE	50	23.0	202.0	499.6	480.2	476.7	414.6	408.2	66.3
45	2	4	HDPE	75	33.0	199.2	672.8	651.9	641.5	564.3	556.1	140.2
46	2	4	PP-R	25	9.0	231.5	121.9	128.0	132.9	119.0	118.0	87.2
47	2	4	PP-R	50	20.0	230.5	159.2	156.5	163.0	150.7	146.2	114.6
48	2	4	PP-R	75	31.0	229.6	207.5	204.2	200.3	184.0	175.7	133.1
49	2	4	PP-R	100	41.0	224.9	261.9	262.9	258.0	225.8	218.1	156.6
50	2	4	PP-R	125	51.0	222.0	340.5	343.1	342.7	298.4	284.0	192.4
51	2	4	PP-R	150	62.0	219.8	423.9	422.3	434.4	380.8	364.3	223.9
52	2	4	PP-R	175	71.5	217.4	547.1	547.9	564.8	503.3	486.2	265.8
53	2	4	PP-R	200	83.5	212.5	685.7	679.2	709.8	641.8	621.8	312.0
54	3	3	HDPE	25	15.0	196.4	197.5	174.6	181.9	133.1	139.6	49.4
55	3	3	HDPE	50	30.0	193.6	298.8	274.9	268.4	195.3	209.0	70.9
56	3	3	HDPE	75	46.0	186.3	459.9	427.0	422.3	336.8	361.3	132.7
57	3	3	HDPE	100	59.5	173.7	617.6	586.2	578.1	472.9	511.5	198.4
58	3	3	PP-R	50	23.0	225.6	26.8	35.2	28.0	17.2	25.2	0.4
59	3	3	PP-R	100	46.0	217.2	115.8	127.4	116.3	82.7	96.9	9.4
60	3	3	PP-R	150	69.0	211.7	529.9	543.2	532.2	470.9	500.4	168.3
61	3	3	PP-R	200	93.0	207.9	697.2	714.9	702.3	622.7	663.0	213.2
62	3	4	HDPE	25	14.0	197.7	238.0	208.5	207.8	149.4	153.4	47.0
63	3	4	HDPE	50	29.0	194.0	371.6	319.1	314.5	218.4	225.3	69.5
64	3	4	HDPE	75	44.0	188.8	541.5	475.8	478.1	362.0	373.2	109.5
65	3	4	HDPE	100	58.0	186.0	697.7	613.8	621.3	490.1	501.3	157.3
66	3	4	PP-R	50	23.0	227.0	33.5	34.8	34.7	26.0	34.7	18.4
67	3	4	PP-R	100	46.0	218.4	143.5	122.7	128.5	81.9	93.6	32.2
68	3	4	PP-R	150	68.0	216.1	305.6	271.1	287.9	222.7	234.0	73.7
69	3	4	PP-R	200	93.0	213.9	459.8	427.8	452.5	391.1	401.2	148.9
70	3	4	PP-R	250	100.5	214.6	542.8	517.8	550.0	484.0	502.3	185.3
71	3	4	PP-R	300	104.0	216.4	438.4	434.5	472.9	421.7	450.7	163.4
72	3	4	PP-R	350	110.5	219.3	400.9	405.1	434.7	402.8	427.8	167.3

**Table A2:** Nomenclature.

a	width of transition	$N_{off}$	number of offset elements
$a_{t}$	temperature-shift factor	$N_{z}$	number of down-channel elements
$D_{b}$	barrel diameter	p	pressure
$D_{s}$	outer screw diameter	$p_{out}$	outlet pressure
e	flight width	**p**	pressure vector
$e_{a}$	flight width (main flight)	t	screw pitch
$e_{b}$	flight width (barrier flight)	T	temperature
*h*	channel depth	$T_{0}$	reference temperature
$h_{f}$	channel depth (feeding zone)	w	channel width
$h_{m}$	channel depth (metering zone)	$w_{1}$	channel width (subchannel 1)
i	number of parallel screw flights	$w_{2}$	channel width (subchannel 2)
k	linearized die conductance	α	temperature coefficient
**K**	conductance matrix	$\delta_{b}$	clearance of barrier flight
L	axial length	$\delta_{f}$	clearance of main flight
*L*_1_	axial start of wave zone	$\dot{\gamma }$	shear rate
*L*_2_	axial end of wave zone	Γ	melt content
$\dot{m}$	mass-flow rate	$\eta_{c}$	viscosity (Carreau-Yasuda model)
${\dot{m}}_{0}$	mass-flow rate (at inlet)	$\eta_{0}$	zero-shear viscosity
**$\dot{m}$**	mass-flow vector	$\eta_{\infty }$	infinite-shear viscosity
${\dot{m}}_{d}$	theoretical drag flow	κ	mixing parameter
**${\dot{m}}_{d}$**	drag-flow vector	λ	characteristic relaxation time
${\dot{m}}_{f}$	cross-channel element flow rate	$\Pi_{p,z}$	dimensionless pressure gradient
${\dot{m}}_{p}$	pressure flow	$\Pi_{V}$	dimensionless volume-flow rate
$n_{c}$	power-law index (Carreau-Yasuda model)	$\rho_{m}$	melt density
n	power-law index	$\varphi_{b}$	pitch angle
N	screw speed		
